# Programmable Axial–Radial Multichannel Nickel Microtubes Fabricated by Core-Filament Jet Electrodeposition

**DOI:** 10.3390/mi17101185

**Published:** 2026-10-13

**Authors:** Wei Wang, Hongtao Qian, Pengju Liu, Zhonghe Li, Yulong Li, Xiaohong Yang

**Affiliations:** 1 School of Mechanical Engineering, Henan Institute of Technology, Xinxiang 453003, China; 2 Henan Provincial Engineering Research Center for Special Energy-Field Manufacturing of Aerospace Critical Components, Henan Institute of Technology, Xinxiang 453003, China; 3 School of Intelligent Manufacturing, Henan Institute of Information Science and Technology, Hebi 458030, China

**Keywords:** jet electrodeposition, hollow metallic microtube, sacrificial fiber templating, segmented electrodeposition, in situ template assembly, electrochemical micromanufacturing

## Abstract

Programmable hollow metallic microdevices with lateral outlets are attractive for distributed fluid handling, but integrating an axial lumen, side channels, and a sealed distal tip remains fabrication-intensive. Here, a core-filament jet electrodeposition (CF-JED) strategy combines segmented electrolyte-jet operation with sacrificial nylon-fiber templating to fabricate multichannel nickel microtubes. An 80 μm nylon core defines the axial lumen, whereas 50 μm transverse and helical fibers prescribe lateral channels. Interrupting the jet creates the in situ assembly interval for template placement; resumed localized electrodeposition encapsulates the fibers and can close the distal tip, and physical pull-out releases the channels without chemical dissolution or drilling. Stable growth was obtained at 2.5 mA with an axial growth rate of approximately 15 μm·min^−1^. Micro-computed tomography revealed a 50 ± 5 μm radial channel and a 76 ± 6 μm axial lumen interconnected within the sealed-tip structure. SEM/EDS confirmed open lateral apertures and nickel-rich walls, while water-immersion gas testing provided qualitative evidence of channel interconnectivity. CF-JED therefore provides a template-defined route to programmable multichannel hollow-metal microstructures; direct liquid-flow, mechanical, and biological validation remain necessary for application-specific use.

## 1. Introduction

Hollow metallic microstructures with internal channels are relevant to microfluidic handling, sensing, and selected biomedical delivery or sampling concepts [1,2,3,4,5,6]. Unlike solid microstructures, hollow devices contain an internal lumen that supports active and metered fluid transport from a reservoir or external pressure source. Conventional hollow designs commonly rely on a terminal outlet, which concentrates release near the distal tip and may be vulnerable to partial obstruction during use. Distributed side-wall outlets can therefore provide alternative lateral transport paths and broaden the region over which fluid distribution or sampling can occur [7,8].

Current hollow-microdevice platforms include metallic and silicon structures [7,8], porous microneedles [9], and polymer devices fabricated by molding or additive manufacturing [10,11,12,13,14]. For metallic or silicon devices, lateral openings are often introduced by laser processing, wet/dry etching, cutting, or other post-processing steps, which can increase process complexity and constrain three-dimensional channel placement [7,8,15]. Sacrificial-template approaches offer another route to internal voids and channels, but their usefulness depends on template placement, removal, material compatibility, and replication fidelity [16]. Notably, sacrificial micromolding combined with selective electrodeposition has enabled hollow metal microneedles for intradermal injection, demonstrating the compatibility of electrodeposition with biomedical hollow-device fabrication [17]. However, coordinated formation of an axial lumen, deliberately positioned radial or helical side channels, and a sealed distal tip within one localized metal-growth sequence remains difficult to accomplish.

Electrochemical additive manufacturing (ECAM) provides non-thermal routes to localized metal growth at micro- and nanoscale dimensions. Relevant approaches include jet electrodeposition [18,19], meniscus-confined deposition [20,21,22], FluidFM-assisted printing [23], and other localized electrochemical methods [24,25]. In particular, filament-guided electrolyte–column electrodeposition has recently been used to fabricate hollow nickel microstructures with continuous internal channels [24]. Previous studies have demonstrated selective jet electrodeposition for micro-object fabrication [26,27,28], path-controlled metallic microstructures by localized electrodeposition [29,30,31,32], and the influence of deposition parameters and hybrid templates on forming behavior [33,34]. However, these studies have primarily addressed solid features, coatings, or general microstructure fabrication rather than hollow metallic microdevices with deliberately positioned axial–radial fluidic networks.

**Figure 1:**
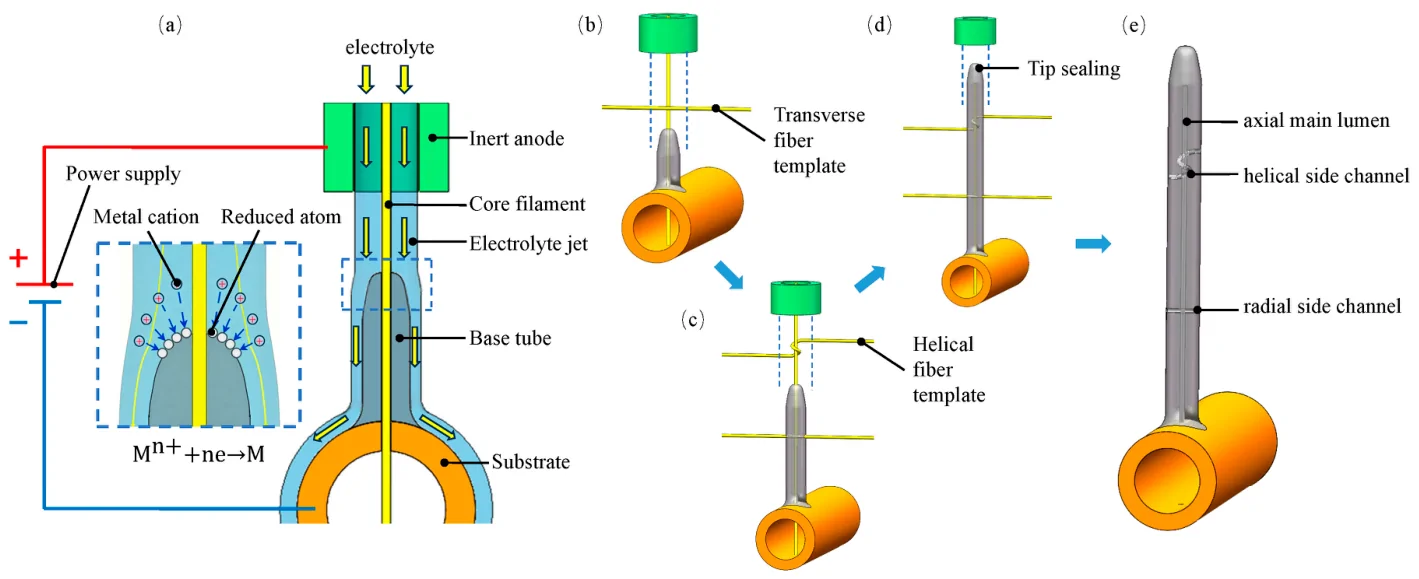
Schematic of core-filament jet electrodeposition (CF-JED) for fabricating multichannel nickel microtubes: (**a**) base-tube formation around the sacrificial core; (**b**) transverse-fiber placement to define a radial side channel; (**c**) helical-fiber placement; (**d**) electrodeposition-mediated template encapsulation and distal-tip sealing; and (**e**) physical pull-out of the core and side-channel templates to reveal the axial lumen and lateral channels. Schematic not drawn to scale.

Here, we present a core-filament jet electrodeposition (CF-JED) strategy that combines segmented electrolyte-jet operation with sacrificial nylon-fiber templating, as shown in Figure 1. Relative to the earlier continuous FG-ECD process [24], CF-JED introduces three coordinated capabilities: (i) a jet-off in situ assembly interval for side-template placement, (ii) template-defined radial and helical lateral-channel layouts, and (iii) resumed encapsulation with integrated distal-tip sealing within the same deposition sequence. An insulating core filament defines the axial lumen, while transverse or helically arranged microfibers define lateral channels. Micro-computed tomography (micro-CT), scanning electron microscopy (SEM), energy-dispersive X-ray spectroscopy (EDS), nanoindentation, and qualitative gas-flow testing were used to assess forming behavior, dimensional fidelity, channel morphology, composition, and network interconnectivity. The present work is positioned as a manufacturing and microfluidic proof-of-concept for programmable hollow metallic microstructures. Skin insertion, biocompatibility, and direct experimental liquid pressure–flow validation are outside the scope of this study and are treated as requirements for future application-specific validation.

## 2. Materials and Methods

### 2.1. Materials and Electrolyte Solution

The electrolyte solution was an aqueous nickel sulfamate bath containing 300 g·L^−1^ Ni(NH_2_SO_3_)_2_·4H_2_O, 15 g·L^−1^ NiCl_2_·6H_2_O, 30 g·L^−1^ H_3_BO_3_, and 1 g·L^−1^ sodium saccharin. The pH was adjusted to 4.0 ± 0.2, and the bath temperature was maintained at 50 ± 2 °C during deposition. High-purity deionized water (resistivity ≥ 18.2 MΩ·cm) was used for solution preparation and sample cleaning.

Polyamide nylon filaments with a diameter of 80 μm were used as sacrificial cores for the central axial lumen, while 50 μm nylon filaments were used as templates for radial and helical side channels. A 2 mm diameter copper tube served as the deposition cathode substrate. Before use, the copper substrate was ultrasonically cleaned sequentially in acetone and ethanol to remove surface contamination.

### 2.2. Core-Filament Jet Electrodeposition (CF-JED) Apparatus

The custom-built CF-JED system comprised four functional modules. The coaxial nozzle was fabricated from polytetrafluoroethylene and had a 0.5 mm inner diameter; a chuck centered and fixed the nylon core filament between the nozzle and substrate. A peristaltic pump delivered the electrolyte solution through the annular nozzle gap at 5–15 mL·min^−1^, forming a stable vertical electrolyte-solution column between the nozzle and substrate. Two parallel platinum sheets served as inert anodes; they were positioned 1 mm from the jet axis on both sides, outside the impinging-jet path, so that oxygen bubbles evolved at the anodes rose freely without entering the jet, and no anodically generated bubbles were observed reaching the growth front during stable operation. A direct-current (DC) power supply operated in constant-current mode. A three-axis computer numerical control (CNC) motion stage with 1 μm nominal resolution translated the substrate relative to the nozzle for programmable spatial growth. The nozzle outlet was positioned 2 mm above the substrate at the start of deposition, and this standoff distance was maintained by the CNC stage during vertical growth.

### 2.3. Segmented Fabrication Process

The multichannel microtubes were fabricated in five sequential steps (Figure 1; Videos S1–S3).

Step I: Base-tube formation. An 80 μm nylon core filament was vertically fixed at the center of the electrolyte jet to define the axial lumen. The jet was initiated at 10 mL·min^−1^. After a stable electrolyte-solution column formed, a constant current of 2.5 mA was applied and nickel was deposited around the core filament. Deposition continued until the tube had sufficient height and rigidity for side-template placement.

Step II: Radial-template placement. The electrolyte jet and deposition current were temporarily stopped, making the exposed core segment accessible for template placement. A 50 μm transverse nylon fiber was positioned across the exposed core under optical-microscope guidance to predefine a radial side channel (Video S1).

Step III: Helical-template placement. A 50 μm nylon fiber was wound helically around the exposed core filament or partially deposited tube at a controlled pitch to predefine a helical side channel. Transverse and helical templates could be introduced separately or together depending on the intended channel architecture (Video S2).

Step IV: Template encapsulation and tip sealing. The electrolyte jet and constant current were restarted. Nickel growth proceeded around the core-fiber assembly and progressively embedded the transverse and helical templates in the tube wall. Continued deposition at the terminal region enclosed the core tip and formed an integrated sealed end (Video S3).

Step V: Physical pull-out demolding. After deposition, the core-fiber–metal composite was rinsed with deionized water. The core filament and side-channel templates were then mechanically extracted from the microtube. The extraction procedure was designed to exploit the radial contraction that accompanies tensile elongation of nylon (Poisson effect), thereby reducing interfacial contact during pull-out. Removal of the core generated the axial lumen, while removal of the transverse and helical fibers generated the predefined side channels. No chemical dissolution step was used.

### 2.4. Characterization

Microtube growth was observed with an optical microscope equipped with an industrial camera for real-time in situ recording. Internal channel geometry was characterized non-destructively by micro-CT (RMCT4000, Royma Tech (Suzhou) Precision Co., Ltd., Suzhou, China) at a voxel size of 1.4 μm. Three-dimensional reconstructions were used to evaluate wall morphology, channel diameters, and the spatial relationships among the axial lumen, radial channel, helical channel, and sealed tip (Video S4).

The surface morphology and cross-sectional features of the deposits were examined using field-emission scanning electron microscopy (FE-SEM, SU8010, Hitachi High-Tech Corporation, Tokyo, Japan) at an accelerating voltage of 10 kV, with elemental composition determined via energy-dispersive X-ray spectroscopy (EDS, X-MaxN, Oxford Instruments, Oxford, UK). To investigate the influence of deposition current on the mechanical response, nanoindentation measurements were performed on cross-sections of specimens prepared under varying applied currents. The local elastic modulus and hardness across the microtube wall were characterized by depth-sensing nanoindentation using a nanoindenter (KLA Corporation, Milpitas, CA, USA) fitted with a Berkovich diamond indenter. Indentation was performed at a maximum load of 300 mN with a continuous stiffness measurement (CSM) frequency of 45 Hz, a loading/unloading rate of 10 mN·s^−1^, and a peak-hold time of 10 s. Indents were placed on polished cross-sections at three radial locations across the wall—near the inner edge, at the wall center, and near the outer edge—with three indents per location (nine indents per specimen); hardness and modulus values are reported as mean ± SD.

A water-immersion gas test was used as a qualitative assessment of channel interconnectivity rather than as a quantitative permeability measurement. The copper tube at the microtube root served as the gas inlet: both ends of the copper tube were open, and neither end was sealed. The free end of the copper tube was fixed to a micro-adapter with silicone sealant and a ferrule fitting and connected to a purified compressed-air source, whereas the joint between the copper tube and the electrodeposited microtube was established in situ during deposition. The only intentionally sealed feature of the assembly is the distal tip of the nickel microtube, which was closed by continued nickel deposition (Section 2.3, Step IV). The specimen was immersed in deionized water. Air pressure was varied from 1 to 50 kPa, and the locations of bubble release were recorded to determine whether the axial lumen communicated with the templated side-wall outlets (Video S5).

## 3. Results and Discussion

### 3.1. Process Window, Growth Morphology, and Nanoindentation Response

**Figure 2:**
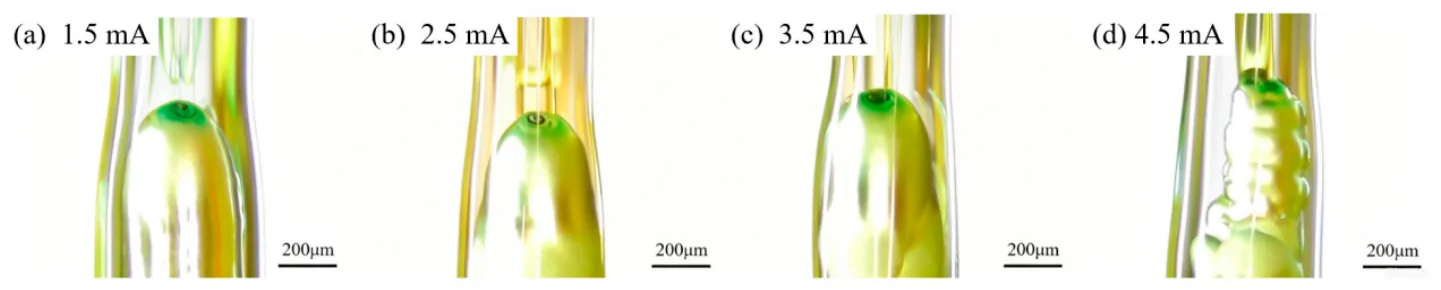
Effect of applied current on nickel growth during CF-JED. In situ optical micrographs show dome-shaped growth at (**a**) 1.5 mA, (**b**) 2.5 mA, and (**c**) 3.5 mA, and nodular growth with visible gas-bubble evolution at (**d**) 4.5 mA. Scale bars, 200 μm.

CF-JED produced directional nickel growth along the nylon core filament and formed hollow microtubes whose morphology depended strongly on applied current. As shown in Figure 2, deposition at 1.5, 2.5, and 3.5 mA maintained a sustained dome-shaped growth front. The axial progression rate of the growth front increased with applied current, being approximately 10 μm·min^−1^ at 1.5 mA, 15 μm·min^−1^ at 2.5 mA, 20 μm·min^−1^ at 3.5 mA, and 35 μm·min^−1^ at 4.5 mA; the corresponding steady-state cell voltages were 3.5, 4.5, 5.5, and 7 V, respectively. At 4.5 mA, the growth front became nodular/cellular and gas bubbles were continuously observed near the deposit tip. This behavior is consistent with intensified cathodic hydrogen evolution and local mass-transport limitation at high polarization. The stationary core can further disturb local ion replenishment, and gas evolution can perturb the current distribution and produce nonuniform growth.

Nanoindentation showed a current-dependent hardness response. The 4.5 mA specimen exhibited the highest measured cross-sectional hardness (4.99 ± 0.22 GPa), but its inner and outer contours were visibly less regular. The 3.5 mA specimen reached 4.66 ± 0.43 GPa, whereas the 1.5 mA specimen exhibited 3.13 ± 0.22 GPa. The 2.5 mA condition provided a practical compromise between morphological stability and deposition rate, maintaining a regular hollow-tube geometry without the pronounced bubble-associated distortion observed at 4.5 mA.

Accordingly, 2.5 mA was used for subsequent multichannel fabrication. Under this condition, the dome-shaped growth front remained stable and visible gas-bubble accumulation near the core was limited. The growth front advanced axially at approximately 15 μm·min^−1^ under this condition, and the wall thickness increased approximately linearly with deposition time.

### 3.2. Template-Defined Construction of Axial–Radial Channels

The defining feature of CF-JED is that the internal channel geometry is prescribed by physical fiber templates rather than generated through stochastic pore formation. The axial lumen is set by the core filament, while the position and trajectory of the side channels are set by the transverse and helical fibers. This separation between geometric definition and metal deposition enables the channel layout to be specified before encapsulation.

**Figure 3:**
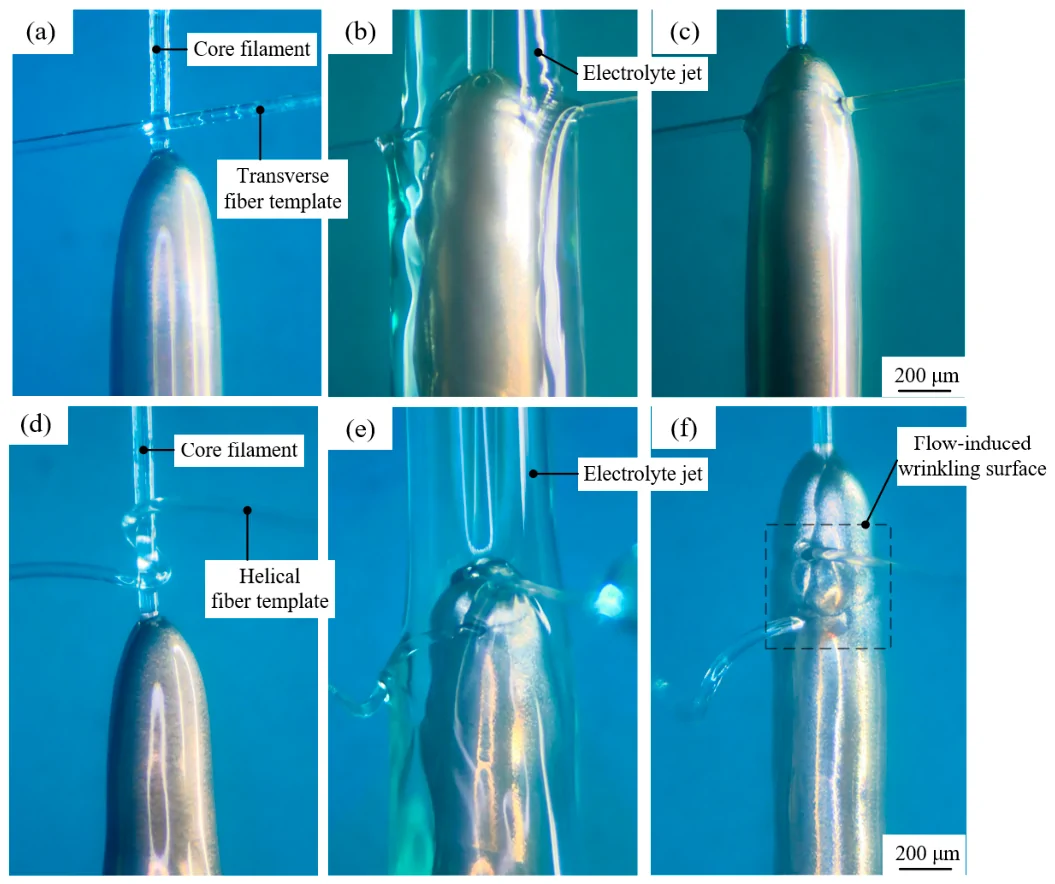
In situ optical observation of fiber templating during CF-JED. (**a**–**c**) Radial-channel construction: (**a**) transverse fiber placed across the exposed core after base-tube formation, (**b**) jet restart and resumed nickel deposition, and (**c**) progressive encapsulation of the transverse template. (**d**–**f**) Helical-channel construction: (**d**) helically wound fiber, (**e**) resumed deposition around the helical core-fiber assembly, and (**f**) localized surface wrinkling observed near the helical template during encapsulation.

After base-tube formation, temporarily stopping the jet and current created a jet-off in situ interval in which a 50 μm transverse fiber could be placed across the exposed 80 μm core (Figure 3a; Video S1). Restarting the jet restored localized nickel growth around the core-fiber assembly (Figure 3b), and continued deposition embedded the transverse template within the tube wall (Figure 3c). A helical template was similarly formed by winding a fiber around the exposed core or partially deposited tube (Figure 3d; Video S2), followed by resumed deposition and encapsulation (Figure 3e,f). The localized surface wrinkling observed near the helical template after resumed deposition (Figure 3f) is tentatively attributed to coupled hydrodynamic and electric-field perturbations around the non-conductive filament: local stagnation/recirculation can alter ion replenishment, while the insulating template can distort the local current distribution. The relative contribution of these effects was not resolved in the present study.

During resumed deposition, the spatially confined electrolyte jet localized the reaction zone around the core-fiber assembly. Constant-current operation provided reproducible growth under a given condition, allowing the embedded fibers to remain identifiable while nickel accumulated around them. Because the intended channel paths were established by the template layout, radial and helical channels could be positioned deliberately rather than relying on random pore connectivity.

After deposition, mechanical extraction of the nylon elements generated the lumen and side channels. Tensile elongation of nylon is expected to be accompanied by radial contraction, which can reduce contact with the surrounding nickel and facilitate pull-out. The observations in this study are consistent with this mechanism, although the local strain, pull-out force and friction during extraction were not measured directly. Micro-CT showed continuous open channel volumes in the reconstructed specimen, and SEM inspection of the accessible channel openings showed no obvious fiber fragments. These observations support successful template evacuation in the imaged geometry but do not establish residue-free removal along the entire internal surface; luminal micro-fracturing or embedded polymer residues along the full channel length cannot be fully excluded. The geometric limits of mechanical extraction, including maximum channel aspect ratio and minimum helical pitch, also remain to be established.

### 3.3. Micro-CT Validation and Dimensional Fidelity

**Figure 4:**
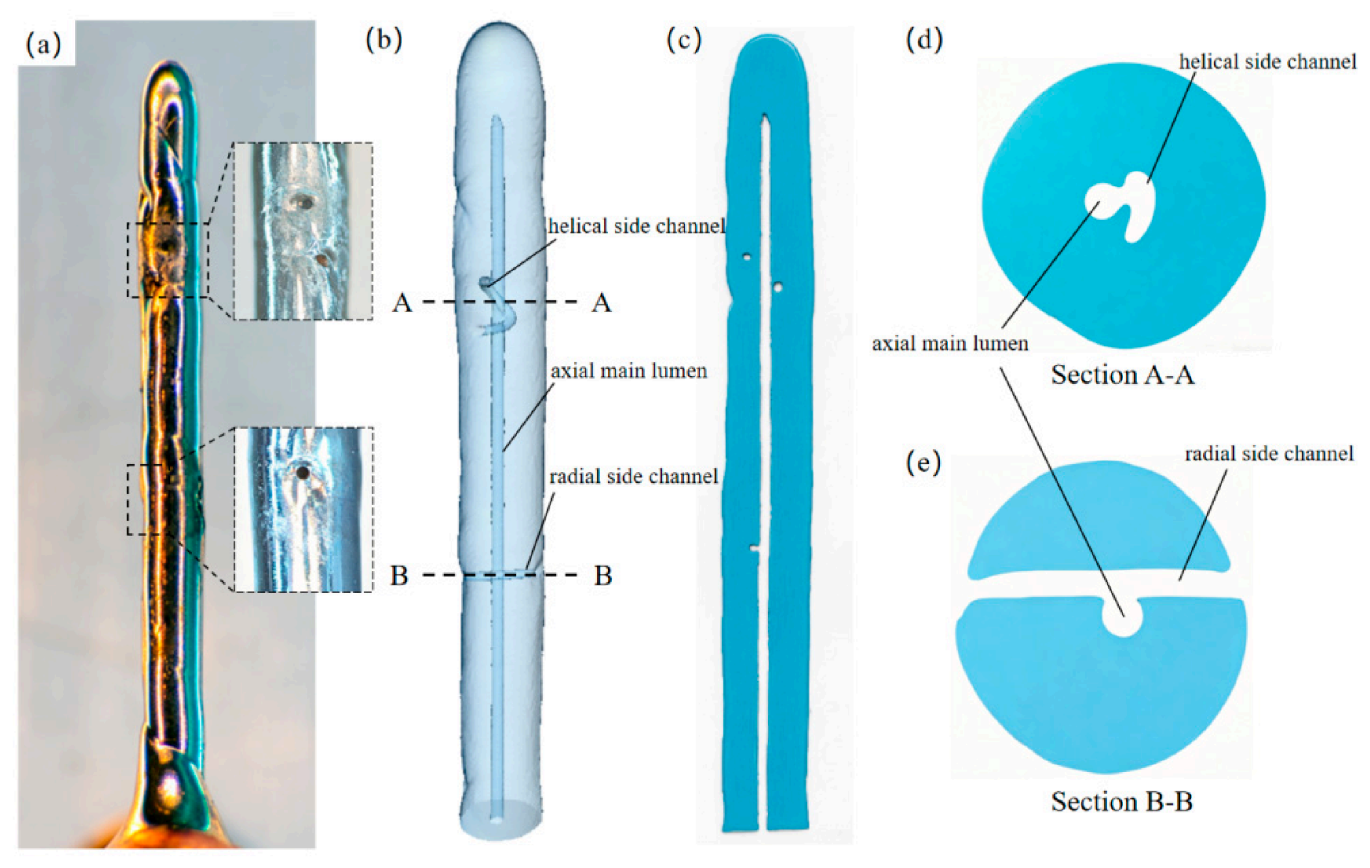
Micro-CT reconstruction of the axial–radial multichannel architecture. (**a**) Overall microtube morphology with magnified side-channel outlet regions. (**b**) Three-dimensional reconstruction showing the axial lumen, helical side channel, and radial side channel, with section planes A-A and B-B indicated by dashed lines. (**c**) Longitudinal section showing the continuous axial lumen and sealed distal region. (**d**) Section A-A through the helical-channel region. (**e**) Section B-B showing the radial channel connected to the axial lumen. Measured radial-channel diameter, 50 ± 5 μm; axial-lumen diameter, 76 ± 6 μm.

Micro-CT reconstruction confirmed the intended axial–radial channel network and sealed distal region (Figure 4; Video S4). The axial lumen extended continuously from the tube root toward the distal tip, where continued nickel deposition produced a closed tip. Sectional reconstructions at different heights showed that the radial and helical channels traversed the tube wall and connected with the axial lumen, forming an interconnected three-dimensional fluidic pathway.

All dimensional values are reported as mean ± standard deviation (SD) of 10 cross-sectional planes measured per channel on one independently fabricated microtube (n = 1); they therefore represent the spread of sectional measurements rather than between-specimen statistics. Dimensional measurements were consistent with replication of the sacrificial templates. The radial channel diameter was 50 ± 5 μm, close to the nominal 50 μm side-template diameter, and the axial lumen diameter was 76 ± 6 μm compared with the 80 μm core filament. The modest difference between nominal and reconstructed diameters is plausibly attributed to three factors: elastic deformation during filament extraction, imaging and segmentation uncertainty, and radial contraction of the nylon core, which was pre-tensioned during fixture alignment and thus elongated before deposition.

The root-mean-square deviation between the reconstructed side-channel positions and the preset fiber coordinates was 42 ± 18 μm. Because template placement was performed manually under optical guidance, this deviation is attributable primarily to assembly accuracy rather than to a programmed robotic placement tolerance. Together, the continuous channel profiles and dimensional agreement support the ability of CF-JED to reproduce predefined fiber layouts, while also identifying manual template positioning as a current source of variability.

### 3.4. SEM/EDS Characterization of Channel Openings and Wall Composition

**Figure 5:**
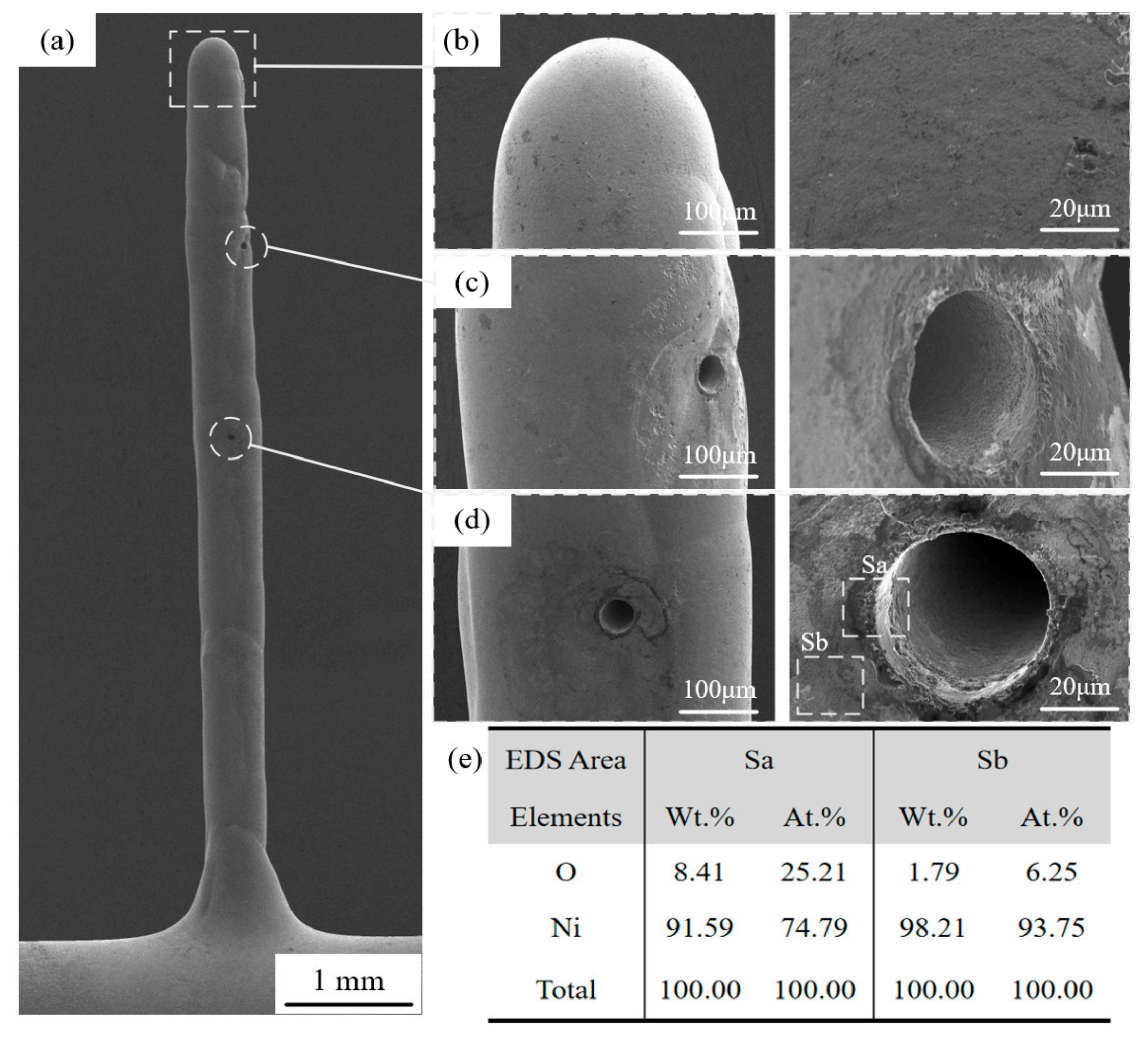
SEM and EDS characterization of the multichannel nickel microtube. (**a**) Low-magnification overview showing the sealed tip and two lateral openings. (**b**) Sealed-tip morphology (**left**) and corresponding surface microstructure (**right**). (**c**) Upper side-channel opening and magnified rim. (**d**) Lower side-channel opening and magnified view, with EDS sites Sa (outer rim) and Sb (inner wall/cross-section). (**e**) Quantitative EDS results: Sa, 91.59 wt% Ni and 8.41 wt% O; Sb, 98.21 wt% Ni and 1.79 wt% O.

SEM confirmed the overall integrity of the fabricated multichannel microtube. The low-magnification image (Figure 5a) shows the tubular structure, sealed distal tip, and two lateral apertures. Higher-magnification images show the sealed-tip morphology (Figure 5b) and the upper and lower side-channel openings (Figure 5c,d).

The side-channel openings had near-circular contours and relatively clean rims. No obvious fiber fragments were visible at the inspected channel edges, which is consistent with successful physical pull-out at those locations. The surrounding nickel displayed a fine granular surface and mild roughness typical of localized electrodeposition under confined transport. Importantly, the visible side-wall openings remained unobstructed for subsequent connectivity testing.

EDS confirmed a nickel-rich deposited wall. At the outer-rim site Sa, the measured composition was 91.59 wt% Ni and 8.41 wt% O, whereas the adjacent site Sb contained 98.21 wt% Ni and 1.79 wt% O. The higher oxygen signal at Sa indicates apparent oxygen enrichment at the analyzed outer-rim region; however, EDS alone cannot establish the presence, chemical state, or origin of an oxide phase. The Sa/Sb comparison should also be interpreted cautiously because the low-energy O Kα signal is more sensitive than the Ni K signal to absorption and take-off geometry in a microscale cavity. The present data therefore do not distinguish oxidation during the jet-off assembly interval from post-process environmental exposure, and the numerical EDS values are treated as semi-quantitative trends rather than an exact compositional depth profile. No organic-rich residue was resolved at the analyzed channel sites; because an instrument-specific calibrated carbon detection limit was not documented in the available records, the absence of a resolved carbon signal is not used as evidence of complete molecular-scale template removal.

### 3.5. Qualitative Gas-Flow Verification of Channel Interconnectivity

**Figure 6:**
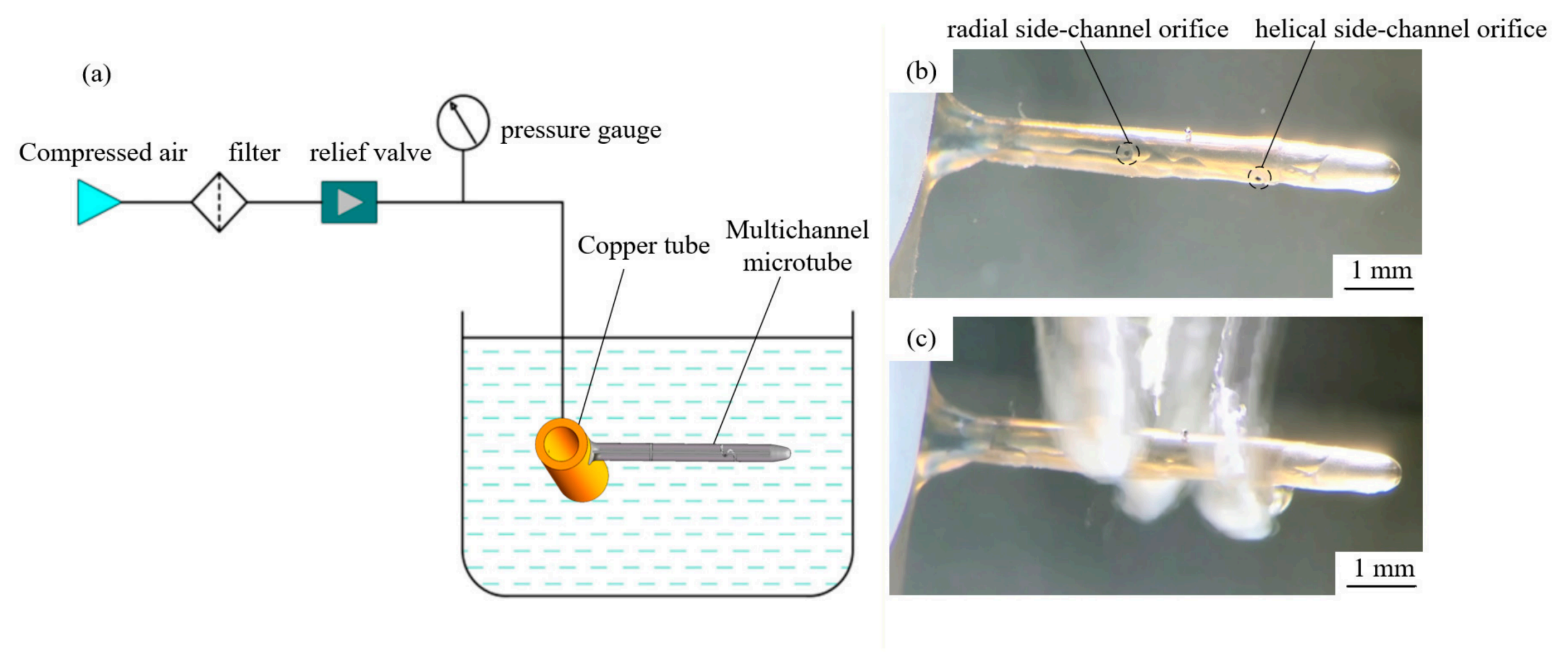
Qualitative gas-flow verification of axial–radial channel interconnectivity. (**a**) Water-immersion test setup in which filtered compressed air is introduced through the microtube root. (**b**) Optical image before pressurization showing the radial and helical side-channel openings. (**c**) Bubble release from the side-wall outlets during gas injection, demonstrating communication between the axial lumen and lateral channels. Note that the micro-adapter and sealing structure at the gas inlet are omitted in the schematic for clarity; the connection details are described in Section 2.4.

The water-immersion gas experiment was used to determine whether the templated channels formed continuous transport paths (Figure 6a; Video S5). Compressed air entered the microtube through the root connection while the specimen was submerged, allowing lateral outlets to be identified from bubble release. Because the gas flow rate was not measured, the experiment provides qualitative connectivity evidence rather than a permeability coefficient or hydraulic resistance.

Before pressurization, the radial and helical side-channel orifices were visible on the tube wall (Figure 6b). During gas injection, bubbles emerged from the predefined side-wall outlets rather than from the sealed distal tip (Figure 6c). This observation is consistent with the micro-CT reconstruction and confirms that the axial lumen communicates with the templated radial and helical channels. The test therefore demonstrates lateral gas transport through the designed network under the tested pressure range.

### 3.6. Design Implications, Limitations, and Comparison with Existing Fabrication Routes

Changing the sacrificial-fiber layout provides distinct channel configurations without changing the basic deposition principle. Discrete radial outlets could be useful where localized lateral release or sampling is desired, whereas a helical channel can extend the lateral flow path and increase the length of wall-associated transport. A sealed distal tip redirects flow toward side-wall outlets and separates lateral delivery from an open-tip design. These are design implications rather than demonstrated biological performance: the present experiments did not evaluate skin penetration, tissue damage, cytocompatibility, sterilization, liquid pressure–flow relationships, or drug-release kinetics.

Relative to representative laser, etching, and additive-manufacturing routes, CF-JED integrates physical channel templating with localized metal growth and distal closure in a coordinated sequence (Table 1). Its principal advantage is deterministic definition of the axial lumen and side-channel trajectories without post-deposition drilling or chemical template dissolution. The current limitations are equally important: side fibers are positioned manually, with placement deviations on the order of tens of micrometers, and fluidic validation remains qualitative. Nickel was used here as a readily electrodepositable model metal for process demonstration; direct tissue-contacting applications would require application-specific material and surface biocompatibility assessment because nickel can cause hypersensitivity in susceptible individuals [35]. Future translation may therefore involve a biocompatible barrier coating or transfer of the CF-JED strategy to another electrodepositable metal or alloy, together with automated fiber placement, quantitative liquid-flow characterization, insertion/mechanical testing, sterilization assessment, and ex vivo or biological validation.

## 4. Conclusions

This study demonstrates a core-filament jet electrodeposition strategy for fabricating nickel microtubes with predefined axial and lateral fluidic channels. Segmented jet operation creates a jet-off in situ interval for side-template placement, after which confined nickel deposition embeds the fiber layout and can seal the distal tip. At the selected 2.5 mA condition, stable growth was obtained, and micro-CT showed radial channels of 50 ± 5 μm and an axial lumen of 76 ± 6 μm. SEM/EDS confirmed well-defined lateral openings and nickel-rich channel walls, while water-immersion gas testing provided qualitative evidence that the axial lumen and side channels are interconnected.

The central contribution is therefore a template-defined physical-templating route for internal channel design in a hollow metallic microdevice, rather than a demonstration of clinical or transdermal performance. Manual template placement and the absence of quantitative liquid-flow, insertion, and biological data currently limit application-level claims. In addition, nickel functions here as a model electrodepositable material and would require material-specific biocompatibility assessment for direct tissue contact. With automated fiber positioning and application-relevant mechanical, fluidic, surface, and biological validation, CF-JED could provide a useful manufacturing platform for future multichannel fluid-distribution and sampling microdevices.

## Figures and Tables

**Table 1:** Comparison of representative fabrication routes for hollow microdevices.

Method	Typical Materials	Side-Channel Control	Tip Sealing	Advantages	Limitations
Laser drilling/cutting [7,15]	Metal, polymer, silicon	High local accuracy; serial openings	Usually a secondary step	Mature and precise	Thermal effects/burrs may occur; serial alignment required
Photolithography /etching [8]	Silicon, glass, polymer	Mask-defined; primarily planar/wafer-based	Often requires additional closure/bonding	High precision and batch compatibility	Material-specific, multi-step processing; constrained 3D freedom
3D printing/molding [10,13,14]	Polymer, resin, hydrogel	High geometric design freedom	Can be incorporated in design	Rapid design iteration and integration	Resolution, material choice, and surface quality depend strongly on process
CF-JED + sacrificial fiber templating (This work)	Nickel (demonstrated); other electrodepositable metals and alloys (potential capability)	Template-defined axial, radial, and helical channels	Integrated by continued deposition	Programmable internal paths; no post-drilling or chemical template dissolution	Manual fiber placement limits throughput and positioning precision; biomedical validation remains necessary

## Data Availability

The data presented in this study are available upon request from the corresponding authors.

## Supplementary Materials

The following supporting information can be downloaded at: https://www.mdpi.com/article/10.3390/mi17101185/s1, Video S1: In situ CF-JED encapsulation of a transverse fiber template—nickel growth around a transverse template defining a radial side channel; Video S2: In situ CF-JED encapsulation of a helical fiber template—nickel deposition over a helically wound template defining a helical side channel; Video S3: In situ formation of a sealed tip by CF-JED—continued nickel growth at the distal region forms a rounded sealed tip; Video S4: Micro-CT reconstruction of the axial–radial multichannel microtube—rotating reconstruction showing the axial lumen, side channels, and sealed tip; Video S5: Water-immersion gas test of lateral channel release—gas bubbles emerge from predefined side-wall outlets.
